# Differentiation of Brain Metastases and Gliomas Based on Color Map of Phase Difference Enhanced Imaging

**DOI:** 10.3389/fneur.2018.00788

**Published:** 2018-09-21

**Authors:** Satoshi Doishita, Shinichi Sakamoto, Tetsuya Yoneda, Takehiro Uda, Taro Tsukamoto, Eiji Yamada, Masami Yoneyama, Daisuke Kimura, Yutaka Katayama, Hiroyuki Tatekawa, Taro Shimono, Kenji Ohata, Yukio Miki

**Affiliations:** ^1^Department of Diagnostic and Interventional Radiology, Osaka City University Graduate School of Medicine, Osaka, Japan; ^2^Department of Medical Physics in Advanced Biomedical Sciences, Faculty of Life Sciences, Kumamoto University, Kumamoto, Japan; ^3^Department of Neurosurgery, Osaka City University Graduate School of Medicine, Osaka, Japan; ^4^Department of Radiological Technology, Osaka City University Hospital, Osaka, Japan; ^5^Philips Japan, Tokyo, Japan

**Keywords:** MRI, PADRE, neuroradiology, neurooncology, astrocytoma, glioma, glioblastoma, metastasis

## Abstract

**Background and objective:** Phase difference enhanced imaging (PADRE), a new phase-related MRI technique, can enhance both paramagnetic and diamagnetic substances, and select which phases to be enhanced. Utilizing these characteristics, we developed color map of PADRE (Color PADRE), which enables simultaneous visualization of myelin-rich structures and veins. Our aim was to determine whether Color PADRE is sufficient to delineate the characteristics of non-gadolinium-enhancing T2-hyperintense regions related with metastatic tumors (MTs), diffuse astrocytomas (DAs) and glioblastomas (GBs), and whether it can contribute to the differentiation of MTs from GBs.

**Methods:** Color PADRE images of 11 patients with MTs, nine with DAs and 17 with GBs were created by combining tissue-enhanced, vessel-enhanced and magnitude images of PADRE, and then retrospectively reviewed. First, predominant visibility of superficial white matter and deep medullary veins within non-gadolinium-enhancing T2-hyperintense regions were compared among the three groups. Then, the discriminatory power to differentiate MTs from GBs was assessed using receiver operating characteristic analysis.

**Results:** The degree of visibility of superficial white matter was significantly better in MTs than in GBs (*p* = 0.017), better in GBs than in DAs (*p* = 0.014), and better in MTs than in DAs (*p* = 0.0021). On the contrary, the difference in the visibility of deep medullary veins was not significant (*p* = 0.065). The area under the receiver operating characteristic curve to discriminate MTs from GBs was 0.76 with a sensitivity of 80% and specificity of 64%.

**Conclusion:** Visibility of superficial white matter on Color PADRE reflects inferred differences in the proportion of vasogenic edema and tumoral infiltration within non-gadolinium-enhancing T2-hyperintense regions of MTs, DAs and GBs. Evaluation of peritumoral areas on Color PADRE can help to distinguish MTs from GBs.

## Introduction

Cerebral metastases and gliomas are major types of brain tumors that are sometimes difficult to discern from one another preoperatively. An important difference between them is the histopathology of non-gadolinium (Gd)-enhancing T2-hyperintense regions. Diffuse astrocytomas (DAs) usually appear as non-enhancing T2-hyperintense lesions (1). On the other hand, metastatic tumors (MTs) and glioblastomas (GBs) are both usually present as ring-enhancing lesions accompanied by non-Gd-enhancing T2-hyperintense peritumoral areas with different microscopic aspects, as pure vasogenic edema is observed with MTs, and both tumoral infiltration and vasogenic edema are observed with GBs (2–4). In fact, several studies have focused on peritumoral areas using advanced MRI techniques, such as diffusion tensor imaging (DTI), perfusion-weighted imaging, magnetic resonance spectroscopy and amide proton transfer imaging (5–8).

The contrast of phase images, which reflects the magnetic permeability of various substances, differs from that of conventional magnitude images (9–12). At present, susceptibility-weighted imaging (SWI) is the most widely used phase-related imaging technique because of its clinical benefits of clear visualization of veins and hemorrhages utilizing the paramagnetic effects derived from deoxyhemoglobin, hemosiderin and non-hem iron (9).

A new phase-related imaging technique called “phase difference enhanced imaging (PADRE)” was recently developed (13–15). PADRE has two strengths over SWI: (i) enhancement of both paramagnetic and diamagnetic substances and (ii) phase selection. For example, vessel-enhanced images (VEI) of PADRE clearly demonstrate veins, hemorrhages and iron-deposits based on paramagnetic susceptibilities, as with SWI. On the other hand, tissue-enhanced images (TEI) of PADRE clearly visualize multiple myelin-rich structures based on diamagnetic susceptibilities, which is not implemented in SWI (13, 16–19). These various enhanced images are post-processed from identical source images, and therefore are free from misregistration. Based on these concepts, we developed a color-map display of PADRE, in which enhanced structures of TEI and VEI are overlaid with different colors to assess multiple structures simultaneously and distinctly.

Previous studies using SWI reported the usefulness of intratumoral susceptibility signals to differentiate MTs from GBs (20–22). However, phase-related imaging techniques have not yet been used to investigate peritumoral areas. Compared with SWI, the color map of PADRE (Color PADRE) contains abundant information regarding normal structures. Therefore, by visualizing internal vessels and myelin-rich structures, Color PADRE may also have the potential to delineate histopathological alterations within non-Gd-enhancing T2-hyperintense regions related to brain tumors, thereby contributing to preoperative diagnosis.

Our goal was twofold. The first was to determine whether preoperative Color PADRE can reflect the histopathology of non-Gd-enhancing T2-hyperintense regions expected from pathological diagnosis of MTs, DAs or GBs. The second was to evaluate the diagnostic potential of Color PADRE to differentiate MTs from GBs, two major brain tumors presenting as ring-enhancing masses accompanied by peritumoral areas.

## Materials and methods

### Subjects

From August 2010 to April 2014, preoperative MR examinations for suspected brain tumors, including the PADRE sequence, were obtained using a 3-T MRI scanner unless there were contraindications for MR examination. The ethics committee of Osaka City University Graduate School of Medicine approved this study. In accordance with the Declaration of Helsinki, written informed consent was obtained from all patients or their legal guardians for the use of clinical data, including MR images, for research purposes. Among the consecutive data mentioned above, patients with pathologically proven MTs, DAs and GBs were retrospectively identified. All pathological diagnoses were confirmed by surgery or biopsy after MRI, according to the 2007 World Health Organization Classification of Tumors of the Central Nervous System (23), the latest criteria available at the time of diagnoses in this study.

**Figure 1:**
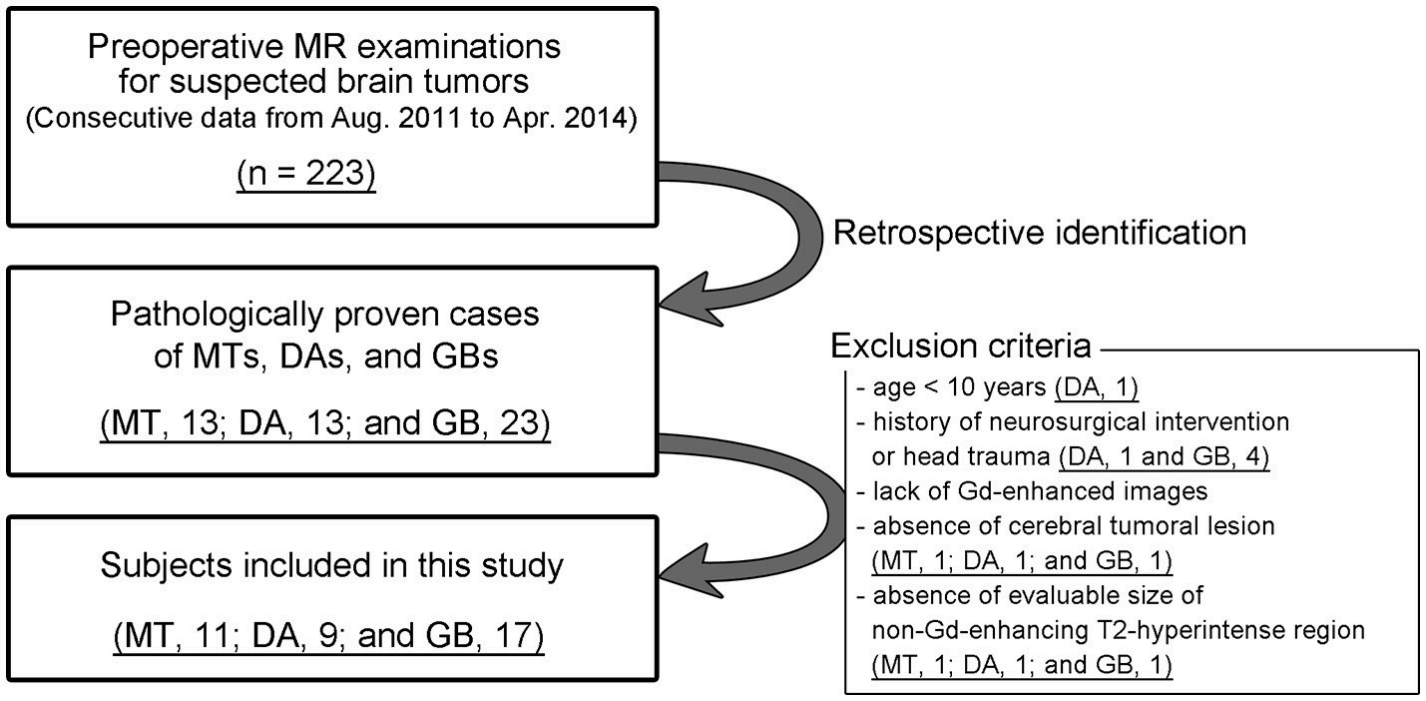
Subject inclusion chart.

Exclusion criteria included the followings: (i) age <10 years; (ii) history of neurosurgical intervention or head trauma; (iii) lack of Gd-enhanced images due to contraindications for Gd-based contrast materials; (iv) absence of cerebral tumoral lesion; and (v) absence of evaluable size (defined as > 5 mm) of non-Gd-enhancing T2-hyperintense regions. The inclusion procedure for the study is shown in Figure 1.

### Imaging protocol

All MR examinations were performed with a 3-T scanner (Achieva; Philips Healthcare, Best, the Netherlands) using an 8-channel head coil. Axial PADRE source images were acquired with three-dimensional principles of echo shifting with a train of observation (3D PRESTO) sequence with the following parameters: repetition time/echo time, 28.1/39.1 ms; field of view, 22 cm; matrix, 512 × 490; flip angle, 10°; slice thickness, 2 mm; slice gap, 1 mm; and image acquisition time, 6 min 50 s. In addition, axial T2-weighted turbo spin echo images (repetition time/echo time, 3,800/80 ms; field of view, 22 cm; matrix, 448 × 326; slice thickness, 5 mm; slice gap, 6.5 mm; image acquisition time, 1 min 46 s) and axial, coronal and sagittal Gd-enhanced fat-suppressed T1-weighted gradient echo images (repetition time/echo time, 5.8/2.6 ms; field of view, 22 cm; matrix, 244 × 244; flip angle, 12°; slice thickness, 3 mm; slice gap, 3 mm; image acquisition time, 5 min 38 s) were obtained as parts of routine examinations.

### Processing of PADRE

The detailed concepts of PADRE processing are reported elsewhere (13–15). Source images of PADRE, acquired with the 3D PRESTO sequence, consisted of pairs of magnitude and phase images. Initially, phase images were unwrapped using a high-pass filter with the homodyne method to remove the effects of static magnetic field. Next, phase (φ, in radian) masks were calculated using the exponential function below with parameters (a, b, and c) adjusted for what to be enhanced. Finally, paired magnitude images and phase masks were multiplied, which resulted in reconstructed enhanced images.

(1) $$f\left(\phi \right)=\;\{\begin{array}{c} e^{-a{\left(\left|\phi \right|\;-\;\frac{b}{100}\pi \right)}^{c}}\;if\;\left|\phi \right|\;\geq \;\frac{b}{100}\pi \;and\\ \{\begin{array}{c} \phi \;>0\;for\;enhancing\;diamagnetism\\ \phi \;<0for\;enhancing\;paramagnetism \end{array}\;\\ 1\;otherwise \end{array}$$

Diamagnetic substances such as myelin show positive phase values whereas paramagnetic substances such as deoxyhemoglobin and hemosiderin show negative phase values in the right-handed system adopted in PADRE. The PADRE technique can produce various types of enhanced images from the same source images by focusing different phases. In the present study, two kinds of enhanced images, TEI and VEI, were reconstructed by substituting a, b, and c with 2, 0, and 1, respectively. PADRE processing was performed off-line with in-house software.

### Creation of color PADRE

**Figure 2:**
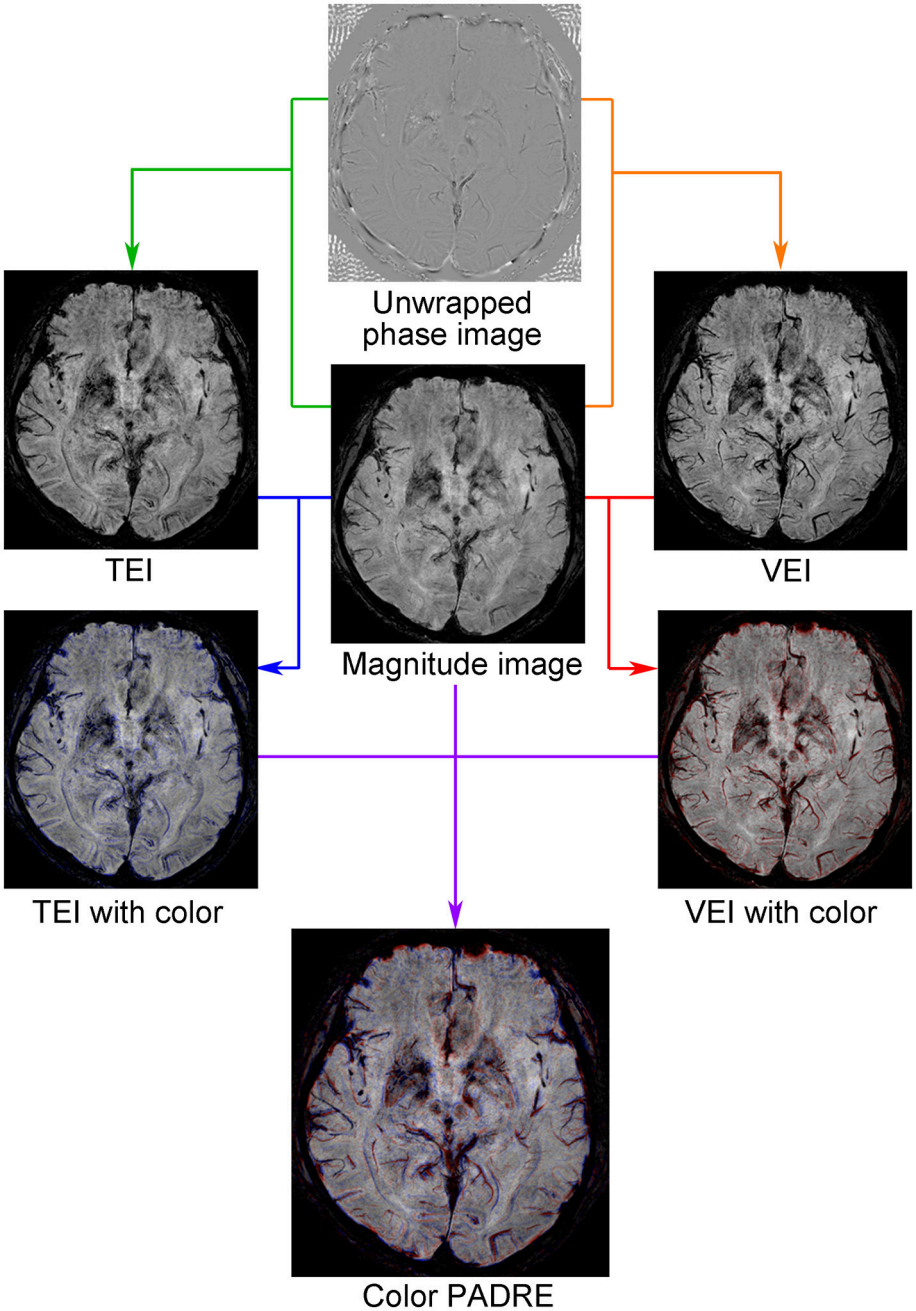
Concept of Color PADRE. TEI, in which myelin-rich structures are enhanced, were post-processed from pairs of magnitude and phase images by focusing positive phases (green line). Similarly, VEI, in which veins, hemorrhages and iron-deposits are enhanced, were post-processed by focusing negative phases (orange line). Structures enhanced on TEI compared with magnitude images were then visualized in blue (blue line) and those enhanced on VEI in red (red line). By combining these and corresponding magnitude images, color maps were created (purple line).

A customized in-house script written in Matlab (version 2016a; Mathworks, Natick, MA, USA) was used for creation of color maps. Structures enhanced with TEI were visualized in blue and those enhanced with VEI in red, and these colors were overlaid on magnitude images containing high-resolution anatomical information (Figure 2). Because TEI, VEI and magnitude images of PADRE were constructed from identical source images, the resultant color maps were free from misregistration, which enabled simultaneous evaluation of myelin-rich structures and veins. In addition, 4 mm-thick minimum intensity projection (MinIP) images of the color maps were generated with DICOM viewer OsiriX MD (version 8.0.2 64-bit; Pixmeo SARL, Bernex, Switzerland) to increase visibility. A distribution version of the software for creating Color PADRE and DICOM files for testing are provided in Supplementary Material.

### Image evaluation

**Figure 3:**
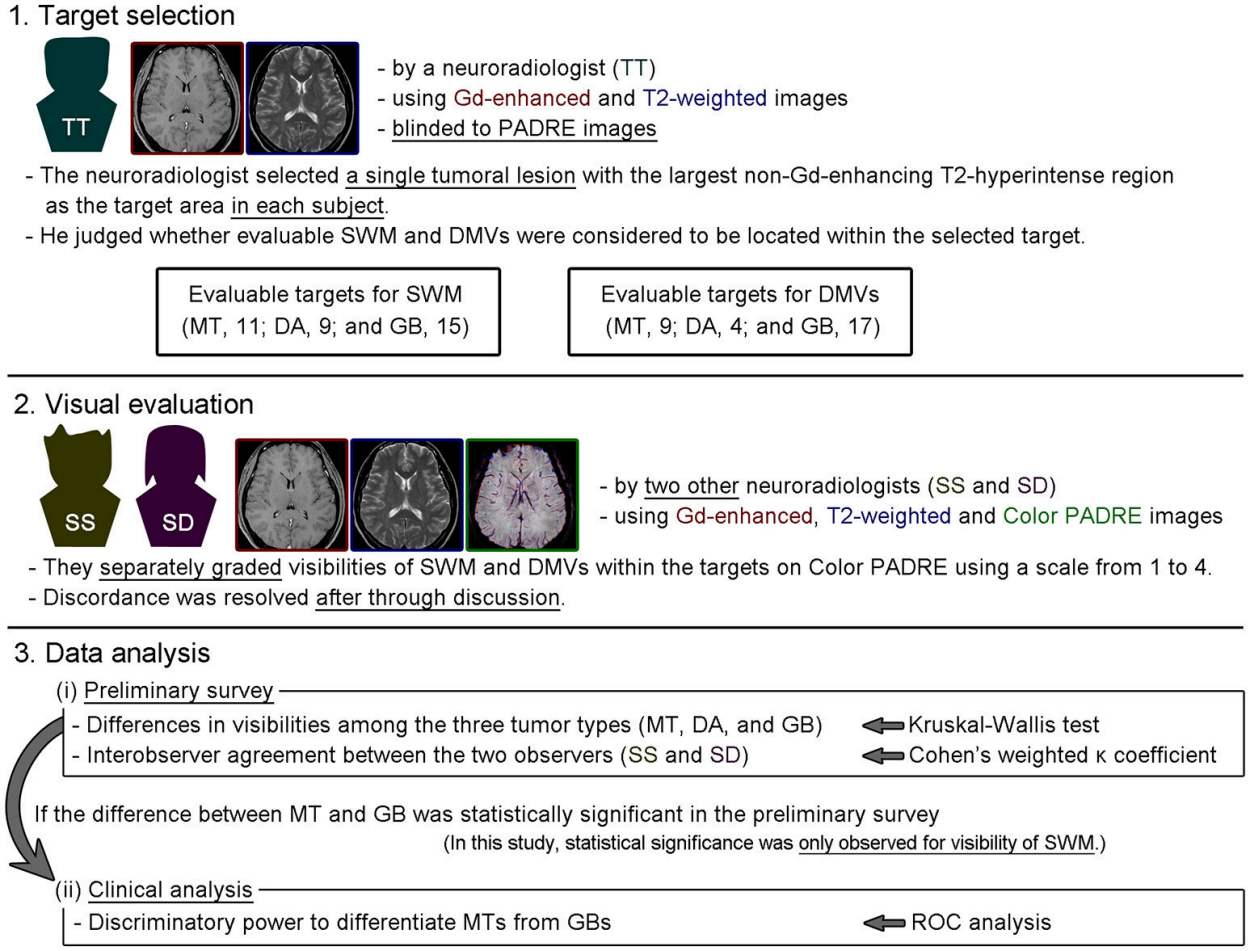
Image evaluation and data analysis strategy adopted in this study.

Our strategy for image evaluation and data analysis is presented in Figure 3.

In the present study, superficial white matter (SWM) and deep medullary veins (DMVs) were evaluated because they are prevalent myelin-rich structures or veins in the brain and are relatively easily recognized on PADRE images. A single tumoral lesion with the largest non-Gd-enhancing T2-hyperintense region was selected as the target in each subject by a neuroradiologist with 7 years of experience, who also judged whether evaluable SWM and DMVs were thought to be located within the target area using Gd-enhanced and T2-weighted images. These procedures were performed with the neuroradiologist blinded to Color PADRE images for minimizing any influence on consequent evaluation.

Then, two neuroradiologists with 11 and 8 years of experience, who were blinded to the pathological diagnoses, separately graded the visibilities of SWM and DMVs within the targets on Color PADRE using Gd-enhanced, T2-weighted and Color PADRE MinIP images. Because there was no dedicated training dataset for this study, the two neuroradiologists briefly viewed the Color PADRE images from all subjects without knowing the pathological diagnoses before commencing evaluation. Cystic or necrotic components were excluded from evaluation. Correction for tumoral location was not performed because there is no established method for correcting locations on PADRE images thus far. The predominant visibilities of SWM and DMVs were scored on an ordinal scale from 1 to 4 (1, poor [<25%]; 2, fair [26–50%]; 3, moderate [51–75%]; and 4, good [>75%]). SWM and DVMs in normal-appearing areas (particularly contralateral corresponding areas) of identical subjects were used as references for grading. The above-mentioned values in percentage were indicated as a guide for scoring. Any discordance in the scores between the two observers was resolved by consensus after through discussion. DICOM viewer OsiriX MD was used for image evaluation.

Two kinds of analyses were performed: (i) a preliminary survey of whether preoperative Color PADRE reflected difference of white matter histology expected from pathological diagnoses of MTs, DAs, or GBs, by comparing the visibilities of SWM and DMVs within the targets among the three groups, and (ii) clinical analysis of the discriminatory power of Color PADRE to differentiate MTs from GBs, if statistical significance between the two tumor types was shown in the preliminary survey.

### Statistical analysis

The two-sided one-way analysis of variance and the chi-squared test were used to compare age and gender distributions. Visibilities of SWM and DMVs among the three groups were assessed using the two-sided Kruskal-Wallis test followed by pairwise comparisons using the Mann-Whitney *U*-test. The Bonferroni-Holm method was adopted for multiple comparisons. Results were considered statistically significant at *p* < 0.05.

Interobserver agreement between the two neuroradiologists was assessed by calculating Cohen's weighted kappa (κ) coefficients. The strength of agreement was judged as follows: κ values of 0.00–0.20, slight; 0.21–0.40, fair; 0.41–0.60, moderate; 0.61–0.80, substantial; and 0.81–1.00, almost perfect (24).

The discriminatory power to differentiate MTs from GBs was assessed by receiver operating characteristic (ROC) analysis, and the Youden index was used to determine the optimal cut-off value.

R (version 3.4.1; R Foundation for Statistical Computing, Vienna, Austria) was used for all statistical analysis.

## Results

Altogether, 13 patients with MTs, 13 with DAs and 23 with GBs underwent preoperative MR examinations, including PADRE. Among them, two patients with MTs (without cerebral lesion, 1; without evaluable size of target, 1), four with DAs (<10 years old, 1; post brain surgery, 1; without cerebral lesion, 1; without evaluable size of target, 1) and six with GBs (post brain surgery or biopsy, 4; without cerebral lesion, 1; without evaluable size of target, 1) were excluded from analysis. Hence, 11 patients with MTs, nine with DAs and 17 with GBs were included in this study. Patient characteristics are shown in Table 1. Patients with DAs were significantly younger than those with other the two tumor types (DA vs. MT, *p* = 0.0010; DA vs. GB, *p* = 0.0010). There was no difference in gender distribution among the three groups (*p* = 0.79). Details of the included MTs were as follows: adenocarcinoma from the lung, 3; clear cell carcinoma from the kidney, 2; small cell carcinoma from the lung, 1; squamous cell carcinoma from the esophagus, 1; adenocarcinoma from the esophagus, 1; adenocarcinoma from the rectum, 1; adenocarcinoma from the breast, 1; and intimal sarcoma from the pulmonary artery, 1.

**Table 1:** Patient characteristics.

		**MT**	**DA**	**GB**
Number		11	9	17
Age (y)	Mean ± SD	63.6 ± 14.2	35.9 ± 14.9	61.5 ± 16.7
	Range	39–84	17–61	29–77
Gender (male/female)		6/5	6/3	9/8

*MT, metastatic tumor; DA, diffuse astrocytoma; GB, glioblastoma; SD, standard deviation*.

**Figure 4:**
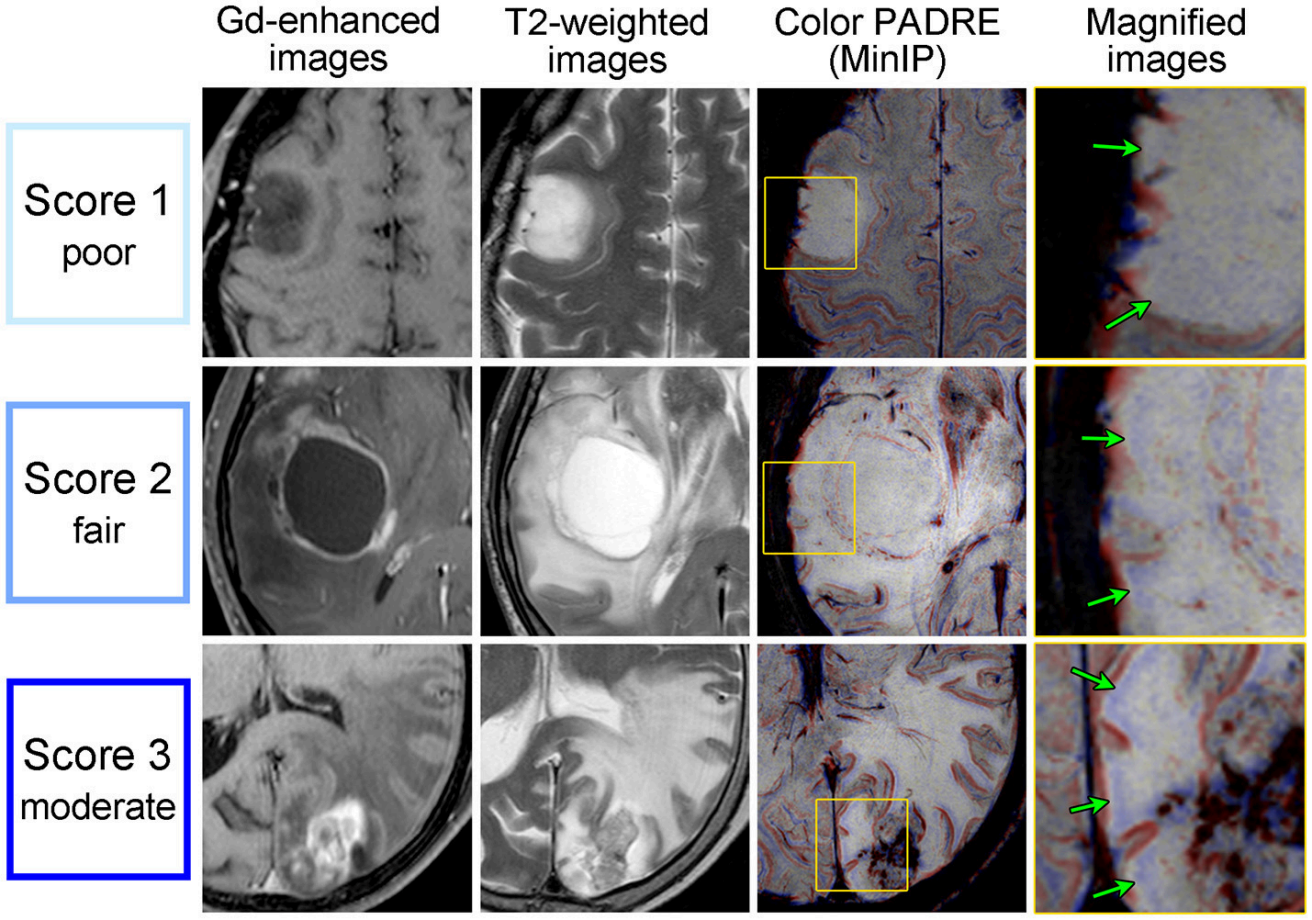
Gd-enhanced images (column 1), T2-weighted images (column 2) and Color PADRE (columns 3 and 4) of the representative cases with scores of 1–3 for SWM. The fourth column shows magnified images of the parts surrounded by yellow squares in the corresponding images in the third column. The first row shows score-1 images of a 43-year-old female with a DA; the second row shows score-2 images of a 67-year-old male with a GB; and the third row shows score-3 images of a 74-year-old female with a MT. No target area received a score of 4 regarding SWM. On Color PADRE, SWM is displayed as blue beneath the cortex. Green arrows in the fourth column indicate the SWM in non-Gd-enhancing T2-hyperintense regions.

**Figure 5:**
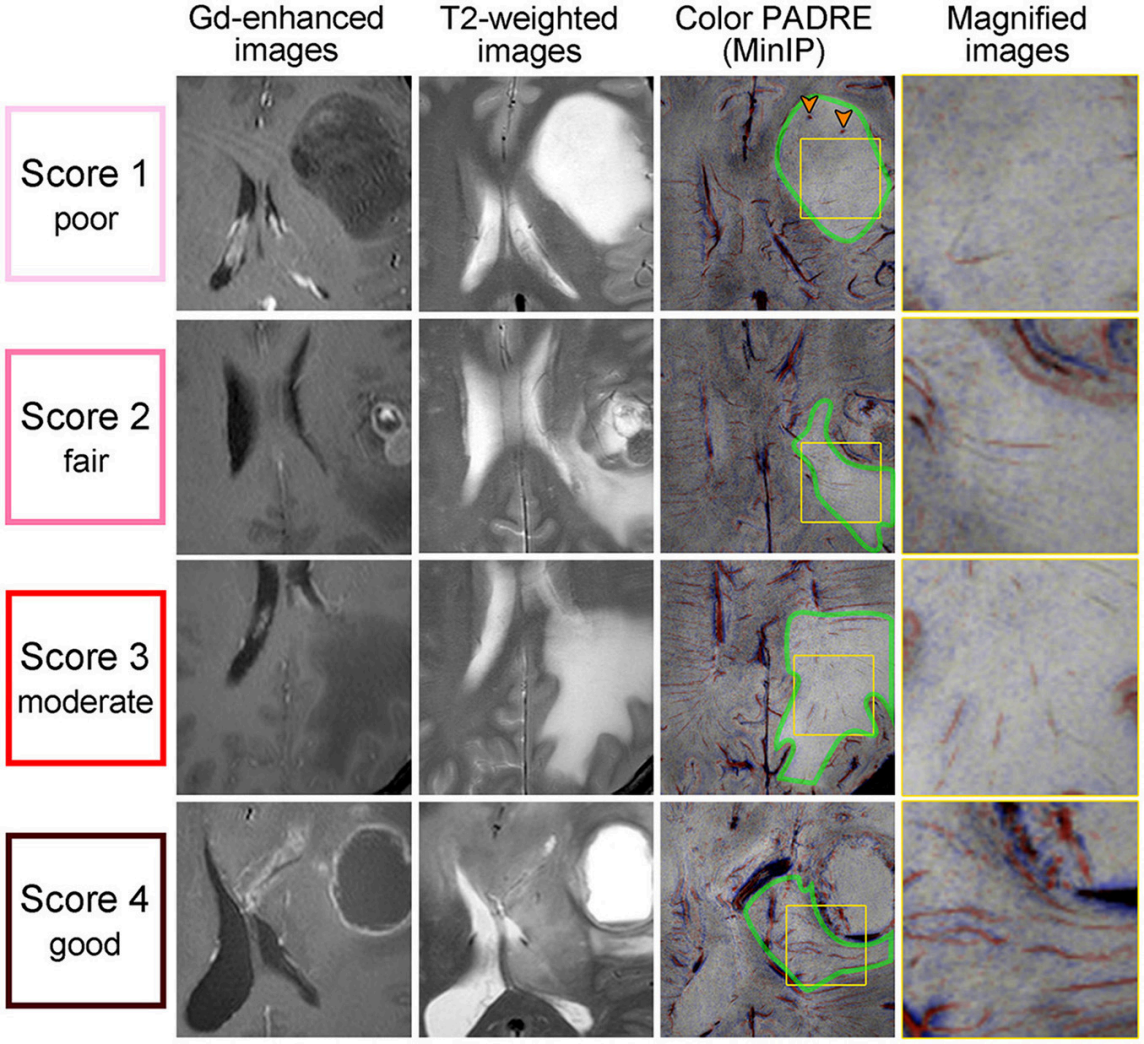
Gd-enhanced images (column 1), T2-weighted images (column 2) and Color PADRE (columns 3 and 4) of the representative cases with DMVs that received scores of 1–4. The fourth column shows magnified images of the parts surrounded by yellow squares in the corresponding images in the third column. The first row shows score-1 images of a 28-year-old female with a DA; the second row shows score-2 images of a 39-year-old male with a MT; the third row shows score-3 images of a 46-year-old male with a MT; and the forth row shows score-4 images of a 31-year-old male with a GB. On Color PADRE, DMVs are displayed as red lines toward the ventricles. Green borders in the third column indicate non-Gd-enhancing T2-hyperintense regions containing DMVs. Orange arrowheads in the third column indicate microhemorrhages.

Figure 4 displays examples of scores 1–3 for SWM and Figure 5 displays those of scores 1–4 for DMVs. No target area received a score of 4 regarding SWM.

**Figure 6:**
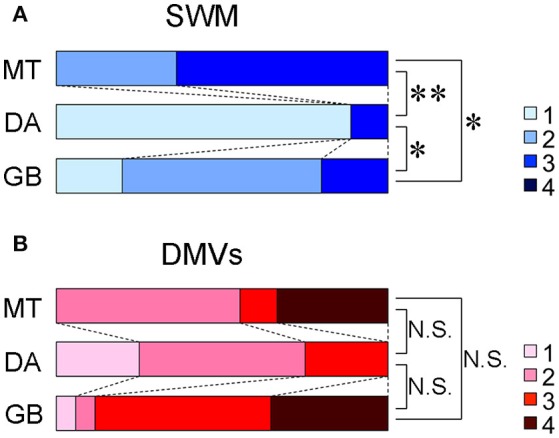
Distribution of the visibilities of SWM **(A)** and DMVs **(B)** on Color PADRE. ^*^p < 0.05, ^**^p < 0.005, N.S. = not significant.

**Figure 7:**
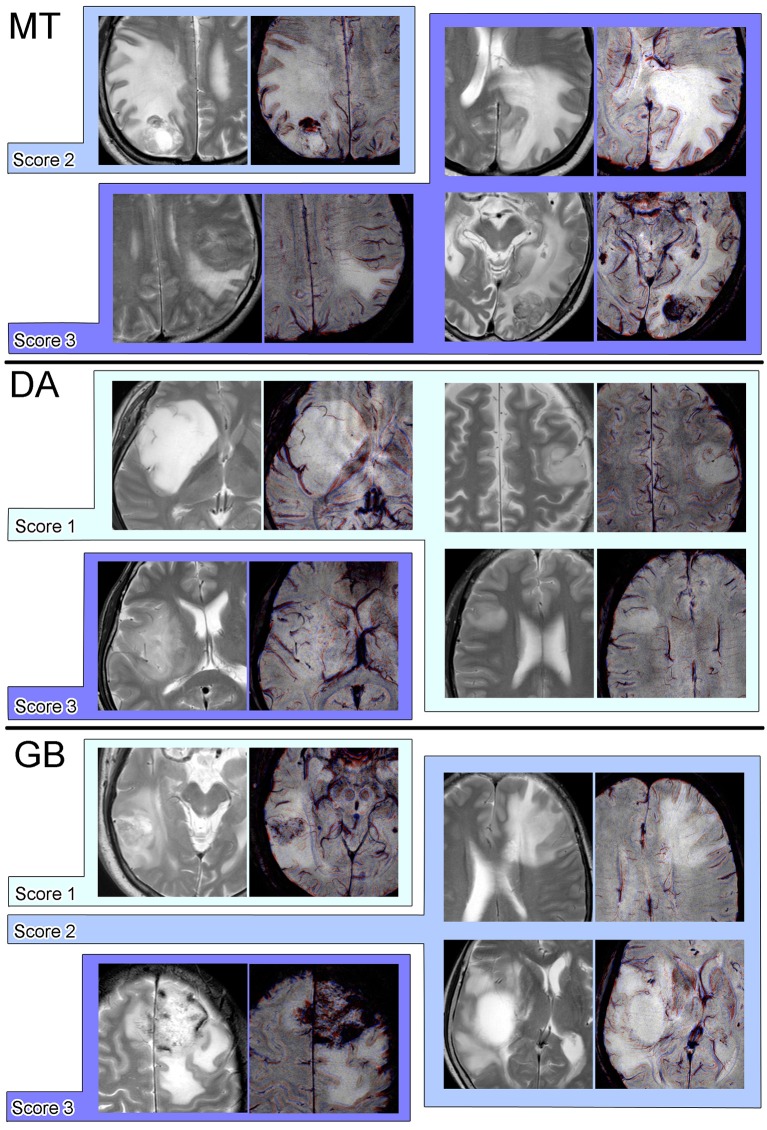
Examples of pairs of T2-weighted and Color PADRE images with scores for SWM on Color PADRE for each tumor type.

A total of 35 targets (MT, 11; DA, 9; GB, 15) and 30 targets (MT, 9; DA, 4; GB, 17) were judged to be evaluable and graded for SWM and DMVs, respectively. The Kruskal-Wallis test indicated that the degree of visibility of SWM was significantly different across the three groups (*p* < 0.001); the score of MT cases was significantly higher than that of GB cases (*p* = 0.017), the score of GB cases was significantly higher than that of DA cases (*p* = 0.014), and the score of MT cases was significantly higher than that of DA cases (*p* = 0.0021) (Figure 6A). Some example Color PADRE images with scores for SWM are presented in Figure 7. On the other hand, differences in visibility were not statistically significant regarding DMVs (*p* = 0.065), although depiction in GBs tended to be better than in the other two tumor types (Figure 6B). Interobserver agreements on the grading of SWM and DMVs were both substantial (κ = 0.65 and 0.75, respectively).

**Figure 8:**
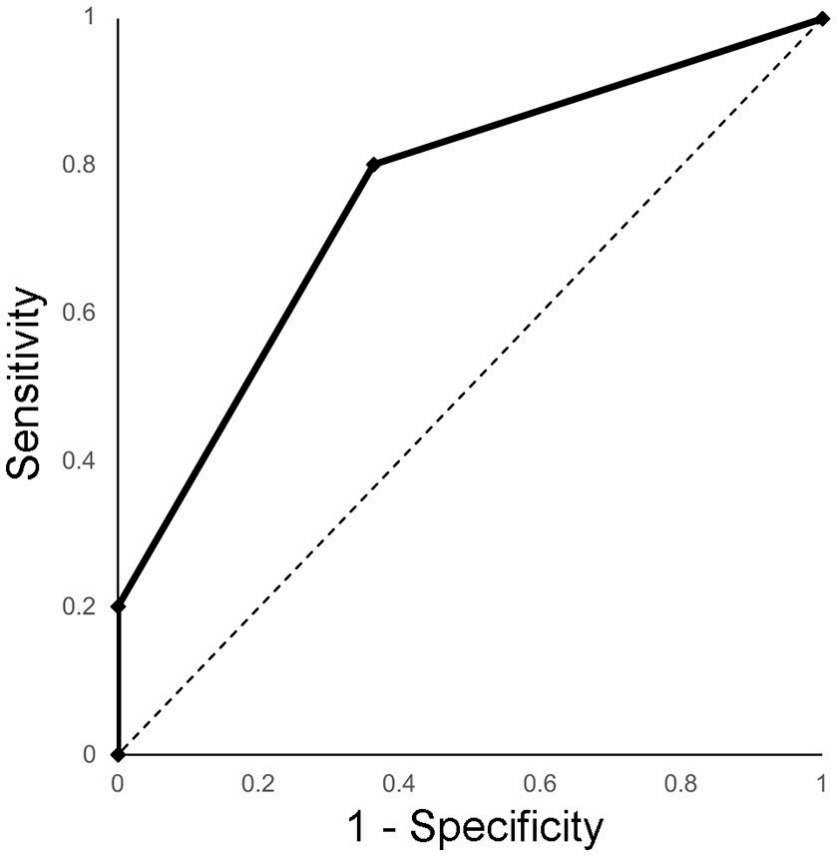
ROC curve of the visibility of SWM on Color PADRE images for distinguishing MTs from GBs.

Because statistical significance between MTs and GBs was shown in the preliminary survey regarding SWM, the discriminatory power of visibility of SWM to discern MTs from GBs was subsequently evaluated. The area under the ROC curve was 0.76 (95% confidence interval, 0.59–0.92) (Figure 8). When the threshold was set between 2 and 3 (GB, ≤ 50% of visibility; MT, >50% of visibility), the diagnostic accuracy, sensitivity and specificity were 71, 80, and 64%, respectively.

## Discussion

Cerebral metastases and gliomas are commonly encountered brain tumors in adults. Distinguishing these tumors, particularly MTs from GBs, is quite important because of the differences in management (25, 26). However, preoperative discrimination is often difficult by conventional MRI, especially if there is no history of known primary malignancy. To date, several studies have focused on peritumoral areas to differentiate cerebral metastases from malignant gliomas. The usefulness of advanced MRI techniques such as DTI (6, 27–33), perfusion-weighted imaging (5, 28, 32, 34–43), magnetic resonance spectroscopy (5, 7, 28, 36, 44–46), amide proton transfer imaging (8) and combinations thereof (47, 48) have been reported.

PADRE is a new phase-related imaging technique that can utilize any phase derived from both paramagnetic and diamagnetic substances. Previous studies of PADRE reported clear demonstration of normal structures, such as small fiber tracts of the brain stem (13), layers of the optic radiation, the stria of Gennari (16, 17), gray-to-white matter interface or SWM (17–19) and the internal structures of the thalamus (14) with TEI. In addition, the relevance of PADRE findings to aging (49) and various pathological conditions including multiple system atrophy of the cerebellar type (13), Parkinson's disease (50, 51), amyotrophic lateral sclerosis (15), multiple sclerosis (52) and Alzheimer's disease (53) has also been reported.

To the best of our knowledge, this is the first published scientific study to investigate whether PADRE offers diagnostic information in clinical cases of brain tumors. We found that the visibility of the SWM in non-Gd-enhancing T2-hyperintense regions was significantly different among MTs, DAs and GBs on Color PADRE. In addition, our results suggested that Color PADRE can discriminate between MTs and GBs by evaluation of SWM in the peritumoral areas.

The peritumoral areas of MTs consist of pure vasogenic edema (2, 54). In contrast, glioma cells invade along existing white matter tracts and T2-hyperintense regions exhibit not only vasogenic edema but also demyelination and other degenerative changes microscopically (4, 55). Even among different types of gliomas, GBs are inferred to have a high proportion of vasogenic edema, as compared to DAs, because of disruption of the blood brain barrier and angiogenesis (4, 56). Tumoral infiltration is considered to induce more severe damage to myelin-rich structures than vasogenic edema. We suppose that the difference in the proportion of vasogenic edema and tumoral infiltration may be reflected in the visibility of SWM on Color PADRE.

On the other hand, the visibility of DMVs was not statistically significant among the three groups. This result suggests that VEI (namely, SWI-like images) alone did not provide diagnostic findings as to non-Gd-enhancing T2-hyperintense regions. However, the visibility of DMVs tended to be greater in GBs than in other two tumor types. GBs are known to be accompanied by angiogenesis (56), which may account for this tendency.

In the present study, interobserver agreements for the visibilities of both SWM and DMVs were not almost perfect, but rather substantial, which can be partly attributed to the non-uniformity of the visibilities of SWM and DMVs within each target area, probably reflecting the heterogeneity of the degrees of edema and tumoral infiltration in addition to the uneven distribution of SWM and DMVs themselves. The low uniformity within non-Gd-enhancing T2-hyperintense regions may be a weakness of evaluation with PADRE.

On PADRE, myelin-rich structures are visualized based on diamagnetic susceptibilities, which are not utilized by other advanced MRI techniques, including DTI (13–15). The usefulness of Color PADRE shown in this study cannot be achieved with other imaging techniques, thus Color PADRE has the potential to yield additional information for preoperative differentiation of MTs from GBs in clinical situations.

There were some limitations to this study. First was the retrospective design and the relatively small sample size. Second was the exclusion of anaplastic astrocytomas from the preliminary analysis due to their histological continuity with DAs and GBs (23). Third was the lack of direct histological correlations of the non-Gd-enhancing T2-hyperintense regions because surgical resection of these areas from all subjects, particularly those with MTs, was ethically difficult. In spite of these limitations, we trust that this study will provide a basis for future prospective and more detailed studies of brain tumors with PADRE.

## Conclusion

Our preliminary data suggest that Color PADRE reflects the suspected histopathology of non-Gd-enhancing T2-hyperintense regions of MTs, DAs and GBs, by yielding novel information based on diamagnetic susceptibilities of myelin-rich structures. Evaluation of peritumoral areas on Color PADRE may contribute to the differentiation of MTs from GBs in clinical situations.

## Data availability statement

The datasets for this manuscript are not publicly available because they contain information that could compromise the privacy of the subjects. Requests to access the datasets should be directed to Satoshi Doishita, sd@med.osaka-cu.ac.jp.

## Author contributions

SD: design of the work; analysis and interpretation of data for the work; and drafting the work; SS: conception and design of the work; acquisition, analysis and interpretation of data for the work; and revising the work critically; TY: design of the work; analysis and interpretation of data for the work; and revising the work critically; TU: design of the work; acquisition and interpretation of data for the work; and revising the work critically; TT: acquisition and analysis of data for the work; and revising the work critically; EY: design of the work; acquisition of data for the work; and revising the work critically; MY: analysis and interpretation of data for the work; and revising the work critically; DK and YK: acquisition of data for the work; and revising the work critically; HT and TS: interpretation of data for the work; and revising the work critically; KO and YM: design of the work; and revising the work critically; All authors: final approval for publication of the content; and agreement to be accountable for all aspects of the work in ensuring that questions related to the accuracy or integrity of any part of the work are appropriately investigated and resolved.

### Conflict of interest statement

MY is an employee of Philips Japan. The remaining authors declare that the research was conducted in the absence of any commercial or financial relationships that could be construed as a potential conflict of interest.

## Abbreviations

PADRE: phase difference enhanced imaging
Color PADRE: color map of phase difference enhanced imaging
MT: metastatic tumor
DA: diffuse astrocytoma
GB: glioblastoma
Gd: gadolinium
DTI: diffusion tensor imaging
SWI: susceptibility-weighted imaging
VEI: vessel-enhanced images
TEI: tissue-enhanced images
3D PRESTO: three-dimensional principles of echo shifting with a train of observation
MinIP: minimum intensity projection
SWM: superficial white matter
DMV: deep medullary vein.
