# Supplementary material for: Differentiation of Brain Metastases and Gliomas Based on Color Map of Phase Difference Enhanced Imaging
Source: Front Neurol. 2018 Sep 21;9:788. doi: 10.3389/fneur.2018.00788 (PMC6160550; doi:10.3389/fneur.2018.00788)
Supplement: Supplementary file 1 [file Data_Sheet_1.PDF]

Supplementary Material

Differentiation of brain metastases and gliomas based on color map of phase difference enhanced imaging

Satoshi Doishita*, Shinichi Sakamoto*, Tetsuya Yoneda, Takehiro Uda, Taro Tsukamoto, Eiji Yamada, Masami Yoneyama, Daisuke Kimura, Yutaka Katayama, Hiroyuki Tatekawa, Taro Shimono, Kenji Ohata, Yukio Miki

*** Correspondence:** Satoshi Doishita: sd@med.osaka-cu.ac.jp; Shinichi Sakamoto: s-sakamoto@med.osaka-cu.ac.jp

1 Supplementary Software Software for creating Color PADRE DICOM, version 1.0 (Windows and macOS).

The software was developed using Matlab and Matlab Compiler (version 2018a, Mathworks, Natick, MA, USA). Please run the installer and follow the instructions to install the software. Internet access is necessary for downloading MATLAB Runtime R2018a.

For Windows: https://doi.org/10.6084/m9.figshare.6958742

For macOS: https://doi.org/10.6084/m9.figshare.6958745

2 Supplementary DICOM files PADRE DICOM files for test.

DICOM files of magnitude, tissue-enhanced, and vessel-enhanced images of PADRE from a test subject are included in a Zip file.

https://doi.org/10.6084/m9.figshare.6879884

3 Supplementary Table 1 Data of all subjects on tumor type and scores for SWM and DMVs.

Data are stored in an Excel file. MT, metastatic tumor; DA, diffuse astrocytoma; GB, glioblastoma; SWM, superficial white matter; DMV, deep medullary vein.

https://doi.org/10.6084/m9.figshare.6875309
